# Description of a new species of *Aplectana* (Nematoda: Ascaridomorpha: Cosmocercidae) using an integrative approach and preliminary phylogenetic study of Cosmocercidae and related taxa

**DOI:** 10.1186/s13071-021-04667-9

**Published:** 2021-03-18

**Authors:** Hui-Xia Chen, Xiao-Hong Gu, Xue-Feng Ni, Liang Li

**Affiliations:** Key Laboratory of Animal Physiology, Biochemistry and Molecular Biology of Hebei Province, College of Life Sciences, Hebei Normal University, 20 East Road of 2nd South Ring, Yuhua District, 050024 Shijiazhuang, Hebei Province People’s Republic of China

**Keywords:** Nematoda, Ascaridomorpha, Systematics, Genetic data, Molecular phylogeny, New species

## Abstract

**Background:**

Nematodes of the family Cosmocercidae (Ascaridomorpha: Cosmocercoidea) are mainly parasitic in the digestive tract of various amphibians and reptiles worldwide. However, our knowledge of the molecular phylogeny of the Cosmocercidae is still far from comprehensive. The phylogenetic relationships between Cosmocercidae and the other two families, Atractidae and Kathlaniidae, in the superfamily Cosmocercoidea are still under debate. Moreover, the systematic position of some genera within Cosmocercidae remains unclear.

**Methods:**

Nematodes collected from *Polypedates megacephalus* (Hallowell) (Anura: Rhacophoridae) were identified using morphological (light and scanning electron microscopy) and molecular methods [sequencing the small ribosomal DNA (18S), internal transcribed spacer 1 (ITS-1), large ribosomal DNA (28S) and mitochondrial cytochrome c oxidase subunit 1 (*cox*1) target regions]. Phylogenetic analyses of cosmocercoid nematodes using 18S + 28S sequence data were performed to clarify the phylogenetic relationships of the Cosmocercidae, Atractidae and Kathlaniidae in the Cosmocercoidea and the systematic position of the genus *Aplectana* in Cosmocercidae.

**Results:**

Morphological and genetic evidence supported the hypothesis that the nematode specimens collected from *P. megacephalus* represent a new species of *Aplectana* (Cosmocercoidea: Cosmocercidae). Our phylogenetic results revealed that the Cosmocercidae is a monophyletic group, but not the basal group in Cosmocercoidea as in the traditional classification. The Kathlaniidae is a paraphyletic group because the subfamily Cruziinae within Kathlaniidae (including only the genus *Cruzia*) formed a seperate lineage. Phylogenetic analyses also showed that the genus *Aplectana* has a closer relationship to the genus *Cosmocerca* in Cosmocercidae.

**Conclusions:**

Our phylogenetic results suggested that the subfamily Cruziinae should be moved from the hitherto-defined family Kathlaniidae and elevated as a separate family, and the genus *Cosmocerca* is closely related to the genus *Aplectana* in the family Cosmocercidae. The present study provided a basic molecular phylogenetic framework for the superfamily Cosmocercoidea based on 18S + 28S sequence data for the first time to our knowledge. Moreover, a new species, *A. xishuangbannaensis* n. sp., was described using integrative approach.

**Graphical abstract:**

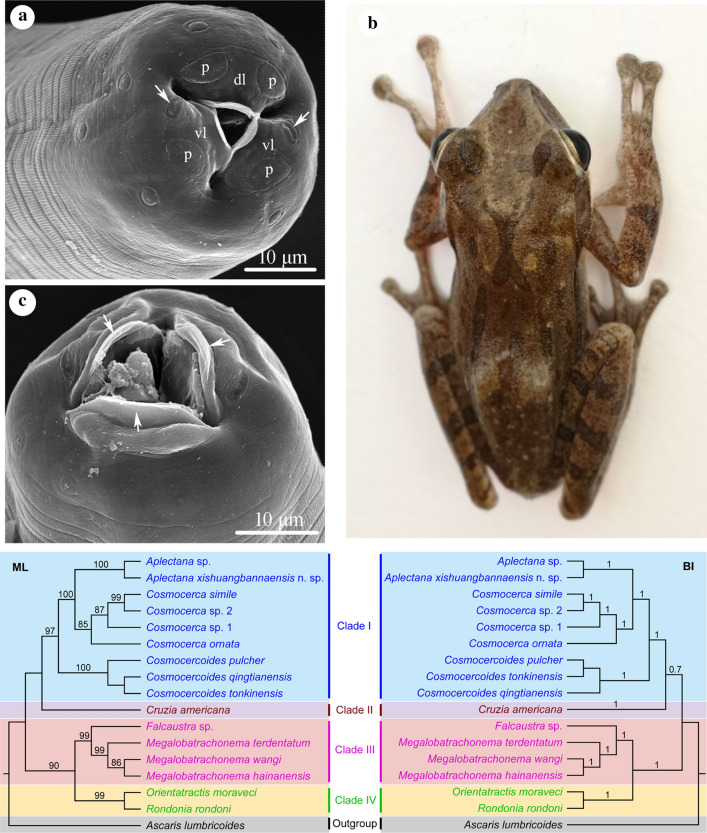

## Background

The superfamily Cosmocercoidea is a group of zooparasitic nematodes and currently comprises three families, namely, Atractidae Railliet, 1917, Cosmocercidae Railliet, 1916, and Kathlaniidae Lane, 1914 [1–3]. Among them, Cosmocercidae is the largest family, including approximately 200 nominal species, which are mainly parasitic in the digestive tract of various amphibians and reptiles worldwide [4–6]. The evolutionary relationships of the Cosmocercidae and the other two families are not yet resolved. Based on morphological and ecological traits, some previous studies [1, 6, 7] considered that the Cosmocercidae represents the ancestral group in Cosmocercoidea.

The present knowledge of the molecular phylogeny of Cosmocercoidea/Cosmocercidae is still very limited. To date, several studies [8–11] have provided molecular phylogenetic analyses to solve the systematic status of some genera in Cosmocercoidea using different genetic data. However, due to the paucity and inaccessibility of suitable material of Cosmocercoidea/Cosmocercidae for genetic analysis, all of these molecular phylogenetic studies have included only small numbers of representatives of these taxa.

To clarify the phylogenetic relationships of the Cosmocercidae and the other families Atractidae and Kathlaniidae in Cosmocercoidea, and the systematic position of the genus *Aplectana* in Cosmocercidae, phylogenetic analyses including the most comprehensive taxon sampling of Cosmocercoidea to date were performed using maximum likelihood (ML) inference and Bayesian inference (BI) based on 18S + 28S sequence data. Moreover, a new species of *Aplectana* was described using an integrative approach.

## Methods

### Parasite collection

A total of 91 *Polypedates megacephalus* (Hallowell) (Anura: Rhacophoridae) collected in the XiShuangBanNa Tropical Botanical Garden, Yunnan Province, China, were investigated for nematode parasites. Nematode specimens were isolated from the intestine of this host and then fixed and stored in 80% ethanol until study.

### Morphological observations

For light microscopical studies, nematodes were cleared in lactophenol. Drawings were made using a Nikon microscope drawing attachment. For scanning electron microscopy (SEM), the anterior and posterior ends of nematodes were re-fixed in 4% formaldehyde solution, post-fixed in 1% OsO4, dehydrated via an ethanol series and acetone, and then critical point dried. Samples were coated with gold and examined using a Hitachi S-4800 scanning electron microscope at an accelerating voltage of 20 kV. Measurements (the range, followed by the mean in parentheses) are given in micrometers (μm) unless otherwise stated. Type specimens were deposited in the College of Life Sciences, Hebei Normal University, Hebei Province, P.R. China.

### Molecular procedures

Genomic DNA from each sample was extracted using a Column Genomic DNA Isolation Kit (Shanghai Sangon, China) according to the manufacturer’s instructions. The partial 18S region was amplified by polymerase chain reaction (PCR) using the forward primer 18S-F (5′-CGCGAATRGCTCATTACAACAGC-3′) and the reverse primer 18S-R (5′-GGGCGGTATCTGATCGCC-3′) [12]. The partial 28S region of nuclear rDNA was amplified by PCR using the forward primer 28S-F (5′-AGCGGAGGAAAAGAAACTAA-3′) and the reverse primer 28S-R (5′-ATCCGTGTTTCAAGACGGG-3′) [13]. The ITS-1 region of nuclear rDNA was amplified by PCR using the forward primer SS1 (5′-GTTTCCGTAGGTGAACCTGCG-3′) and the reverse primer SS2R (5′-AGTGCTCAATGTGTCTGCAA-3′) [14]. The partial *cox*1 region was amplified by PCR using the forward primer COIF (5′-TTTTTTGGTCATCCTGAGGTTTAT-3′) and the reverse primer COIR (5′-ACATAATGAAAATGACTAACAAC-3′) [15]. The cycling conditions were described by the previous study [9]. PCR products were checked on GoldView-stained 1.5% agarose gels and purified with the Column PCR Product Purification Kit (Shanghai Sangon, China). Sequencing was carried out using a DyeDeoxy Terminator Cycle Sequencing Kit (v.2, Applied Biosystems, Foster City, CA, USA) and an automated sequencer (ABI-PRISM 377). Sequencing of each sample was carried out on both strands. Sequences were aligned using ClustalW2. The DNA sequences obtained herein were deposited in the National Center for Biotechnology Information (NCBI) database (http://www.ncbi.nlm. nih.gov) and compared (using the BLASTn algorithm) with those available in the GenBank database.

### Phylogenetic analyses

Phylogenetic trees were constructed based on the 18S + 28S sequence data using maximum likelihood (ML) in IQ-TREE and Bayesian inference (BI) in MrBayes 3.2 [16, 17]. *Ascaris lumbricoides* Linnaeus, 1758 (Ascaridida: Ascaridoidea) was used as the outgroup. The ingroup included 16 cosmocercoid species belonging to 8 genera in 3 different families: Cosmocercidae, Atractidae and Kathlaniidae. The detailed information of nematode species included in the phylogenetic analyses, is provided in Table 1. We used a built-in function in IQ-TREE to select a best-fitting substitution model for the sequences according to the Bayesian information criterion [18]. The TIM3e + G4 model for 18S + 28S sequence data were identified as the optimal nucleotide substitution model. Reliabilities for the ML tree were tested using 1000 bootstrap replications, and the BI tree was tested using 50 million generations, and bootstrap values exceeding 70% were shown in the phylogenetic tree.

**Table 1:** Representatives of Cosmocercoidea used for phylogenetic analyses related to information on host, locality and GenBank ID

Species	Host	Locality	GenBank ID		References
			18S	28S	
*Aplectana xishuangbannaensis* n. sp.	*Polypedates megacephalus* (Hallowell)	China	MW329041	MW329038	Present study
*Aplectana* sp.	*Hylarana spinulosa* (Smith)	China	MW329991	MW364062	Present study
*Cosmocerca ornata* (Dujardin, 1845)	*Hylarana spinulosa* (Smith)	China	MW326676	MW326675	Present study
*Cosmocerca simile* Chen, Zhang, Feng & Li, 2020	*Bufo gargarizans *Cantor	China	MN839758	MN833301	Chen et al. [10]
*Cosmocerca* sp. 1	*Hoplobatrachus chinensis* (Osbeck)	China	MW329987	MW329989	Present study
*Cosmocerca* sp. 2	*Bufo melanostictus* Schneider	China	MW329990	MW329988	Present study
*Cosmocercoides pulcher* Wilkie, 1930	*Bufo japonicus formosus*	Japan	LC018444	LC018444	Tran et al. [46]
*Cosmocercoides qingtianensis* Chen, Zhang, Nakao & Li, 2018	*Bufo gargarizans* Cantor	China	MH178321	MW325956	Chen et al. [47]; Present study
*Cosmocercoides tonkinensis* Tran, Sato & Luc, 2015	*Acanthosaura lepidogaster* (Cuvier)	Vietnam	AB908160	AB908160	Tran et al. [46]
*Cruzia americana* Maplestone, 1930	*Didelphis virginiana* Kerr	USA	U94371	U94757	Nadler and Hudspeth [13]
*Falcaustra* sp._T	*Lithobates catesbeianus* (Shaw); *Indotestudo elongate* (Blyth)	Japan; China	AB818380	MF094270	Hasegawa et al. [48]; Li et al. [49]
*Megalobatrachonema hainanensis* Chen, Zhang & Li, 2019	*Amolops hainanensis* (Boulenger)	China	–	MH545569	Chen et al. [9]
*Megalobatrachonema terdentatum* (Linstow, 1898)	*Lissotriton vulgaris* (Linnaeus)	Germany	–	MN444705	Sinsch et al. [50]
*Megalobatrachonema wangi* Chen, Zhang, Sinsch, Scheid, Balczun & Li, 2020	*Quasipaa exilispinosa* (Liu & Hu)	China	MW325957	MN245660	Present study; Chen et al. [11]
*Orientatractis moraveci* Cavalcante, Silva, Santos, Chagas-Moutinho & Santos, 2016	*Pimelodus blochii* Valenciennes	Brazil	KX524513	KX524514	Cavalcante et al. [51]
*Rondonia rondoni* (Travassos, 1920)	*Pterodoras granulosus* (Doradidae); *Pimelodus blochii* Valenciennes	Peru; Brazil	DQ442679	KX524512	Wijova et al. [52]; Cavalcante et al. [51]
*Ascaris lumbricoides* Linnaeus, 1758	*Homo sapiens* Linnaeus	USA	M74585	U94751	Müller et al. [53]; Nadler and Hudspeth [13]

## Results

Family Cosmocercidae (Railliet, 1916)

Genus *Aplectana* Railliet & Henry, 1916

***Aplectana xishuangbannaensis n. sp.***

***Type host:*** White-spotted thigh tree-frog *Polypedates megacephalus* (Hallowell) (Anura: Rhacophoridae).

***Type-locality:*** XiShuangBanNa Tropical Botanical Garden (21°41′N, 101°25′E), Yunnan Province, China.

***Type specimens:*** Holotype: male (HBNU–N-2020A009L); allotype: female (HBNU–N-2020A010L); paratypes: 41 males, 122 females (HBNU–N-2020A011L).

***Site of infection***: Intestine.

***Prevalence and intensity of infection:*** 12.1% (11 *P. megacephalus* infected out of 91 examined) were infected with intensity of 1–88 (mean 15.0) nematodes.

***ZooBank registration:*** To comply with the regulations set out in Article 8.5 of the amended 2012 version of the International Code of Zoological Nomenclature (ICZN) [19], details of the new species have been submitted to ZooBank. The Life Science Identifier (LSID) of the article is urn:lsid:zoobank.org:pub:09F4B1EF-C3AF42E6-80E6-B734D6B084B8. The LSID for the new name *Aplectana xishuangbannaensis* is urn:lsid:zoobank.org:act:5E4C6C18-7B72-4C28-BD28-6964C6D8F0A3.

***Etymology:*** The specific epithet refers to the type location XiShuangBanNa Tropical Botanical Garden, Yunnan Province, China.

### Description

#### General

**Fig. 1:**
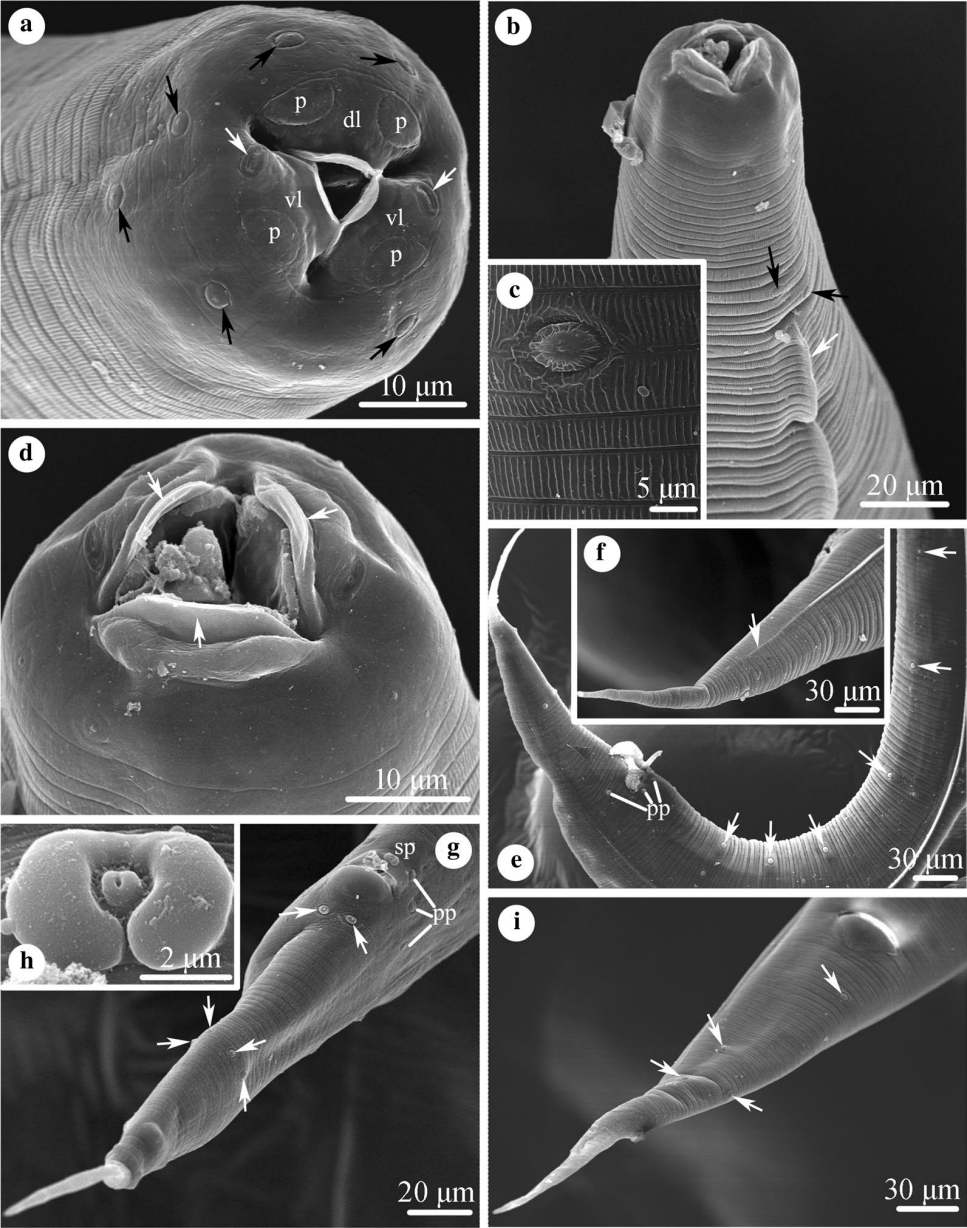
Scanning electron micrographs of *Aplectana xishuangbannaensis* n. sp. collected from *Polypedates megacephalus* (Hallowell) (Anura: Rhacophoridae) in Yunnan Province, China. **a** Cephalic end of female (somatic papillae (black arrows) and amphids (white arrows) arrowed), subapical view. **b** Anterior part of male (somatic papillae (black arrows) and lateral ala (white arrow) arrowed), lateral view. **c** Magnified image of somatic papilla and longitudinal stockade-like ornamentation of cuticle of female. **d** Cephalic end of male (inner flanges arrowed), subapical view. **e** Posterior end of male (precloacal papillae arrowed), lateral view. **f** Tail of male (lateral ala arrowed), lateral view. **g** Tail of male (four pairs of postcloacal papillae arrowed), ventro-lateral view. **h** Magnified image of single, median precloacal papilla. **i** Tail of female (somatic papillae arrowed), ventral view

**Fig. 2:**
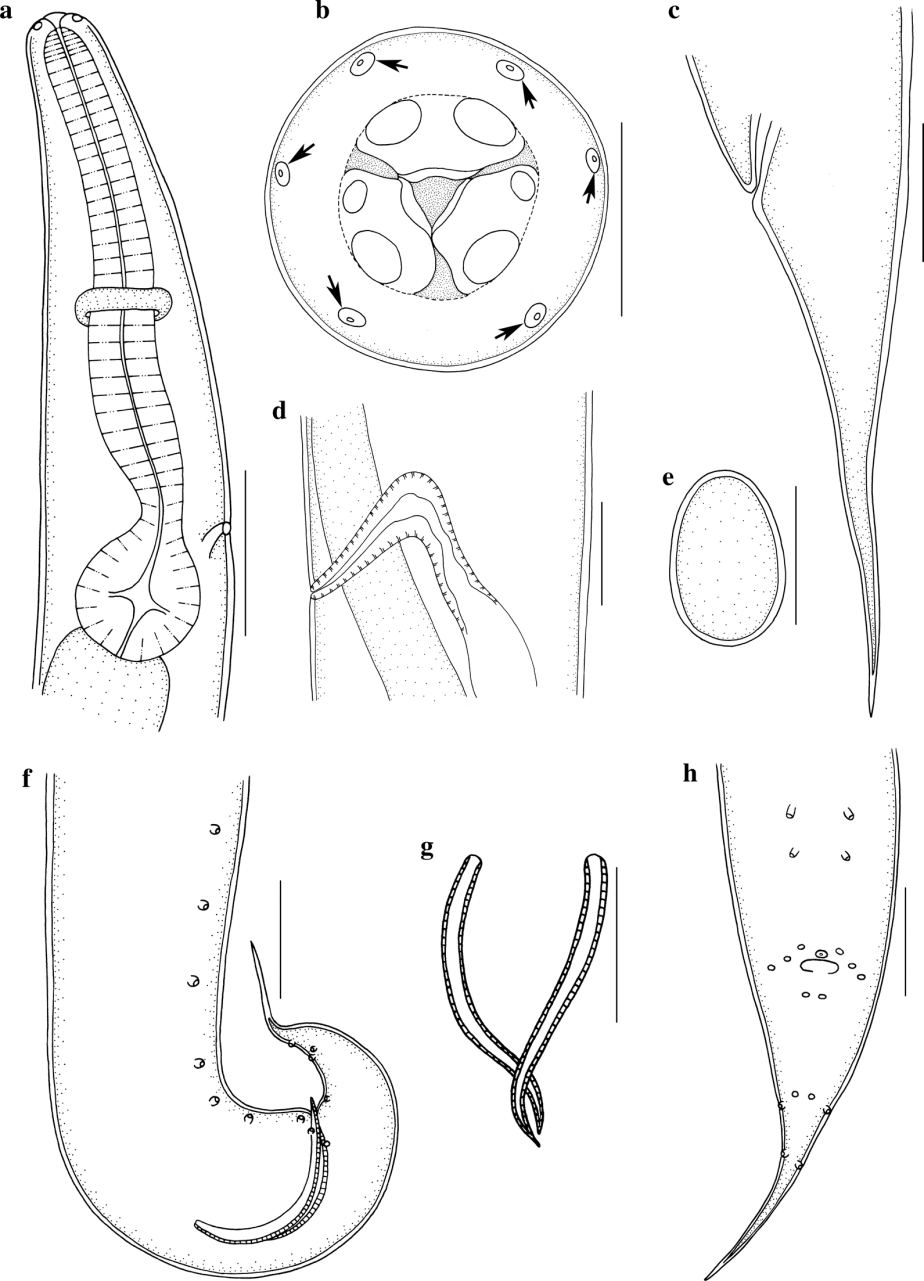
*Aplectana xishuangbannaensis* n. sp. collected from *Polypedates megacephalus* (Hallowell) (Anura: Rhacophoridae) in Yunnan Province, China. **a** Anterior part of male, lateral view. **b** Cephalic end of female (somatic papillae arrowed), apical view. **c** Tail of female, lateral view. **d** Vulva, lateral view. **e** Egg. **f** Posterior end of male, lateral view. **g** Spicules. **h** Tail of male, ventral view. *Scale bars*: **a**, **c**‒**h**, 100 μm; **b**, 20 μm

Small-sized, whitish nematodes. Body cylindrical, maximum width at about region of middle body. Cuticle with fine transverse striations and longitudinal stockade-like ornamentation (Fig. 1a–c). Somatic papillae small, distributed irregularly over body surface (Figs. 1a–c, e, i, 2b). Lateral alae extending from 60–70 posterior to base of lips as far as about middle of tail in both sexes (Fig. 1b, f, i). Oral aperture simple, triangular, surrounded by 3 small lips, each with inner flanges (Figs. 1a, b, d, 2b). Dorsal lip with pair of large double cephalic papillae; subventral lips with single large double cephalic papilla and amphid each (Figs. 1a, 2b). Oesophagus divided into anterior short pharynx, cylindrical corpus, slightly narrower isthmus and terminal posterior bulb with valves (Fig. 2a). Nerve ring located at about 1/2 of oesophageal length. Excretory pore slightly anterior to of oesophageal bulb (Fig. 2a). Tail of both sexes conical, with long filamentous tip (Figs. 1e–g, i, 2c, f, h).

#### Male

[Based on 10 mature specimens; Figs. 1b, d–h, 2a, f–h]: Body 2.32–2.72 (2.49) mm long, maximum width 139–178 (158). Oesophagus 317–426 (374) long, representing 12.6–16.1 (15.0) % of body length; pharynx + corpus + isthmus 248–356 (307) long, bulb 59–69 (67) × 50–59 (54) (Fig. 2a). Nerve ring 158–198 (176) and excretory pore 257–376 (334) from anterior extremity, respectively (Fig. 2a). Posterior end of body distinctly curved ventrally (Figs. 1e, 2f). Spicules small, similar in shape and length, 139–178 (161) long, distal end pointed, representing 5.98–7.09 (6.47) % of body length (Fig. 2g). Gubernaculum absent. Caudal papillae: 6 pairs of precloacal, 3 pairs paracloacal (distinguishable from somatic papillae) and 4 pairs postcloacal papillae. Single median, ventral precloacal papilla present (Figs. 1g, h, 2h). Tail 198–248 (230) long, representing 8.26‒9.84 (9.26) % of body length (Figs. 1e–g, 2f, h).

#### Female

[Based on 10 mature specimens; Figs. 1a, c, i, 2b–e]: Body 3.54–3.86 (3.65) mm long, maximum width 248–297 (272). Oesophagus 416–446 (431) long, representing 11.0–12.6 (11.8) % of body length; pharynx + corpus + isthmus 347–366 (356) long, bulb 69–79 (74) × 50–69 (62). Nerve ring 208–228 (215) and excretory pore 347–386 (366) from anterior extremity, respectively. Vulva transverse slit, 1.60–2.10 (1.89) mm from anterior extremity, at 44.8–54.5 (51.8) % of body length. Ovaries two, located anterior to vulva. Vagina muscular (Fig. 2d). Uteri amphidelphic, full of eggs in different stages of development; egg oval, large, with smooth surface, 149–297 (205) × 99–238 (146) (*n* = 20) (Fig. 2e). Tail 347–406 (384) long, representing 9.78‒11.1 (10.5) % of body length (Figs. 1i, 2c).

### Genetic characterization

#### Partial 18S region

Three 18S sequences of *Aplectana xishuangbannaensis* n. sp. (accession numbers MW329041–MW329043) obtained were all 1539 bp long, representing only one genotype. There is no species of *Aplectana* with 18S sequenced registered in GenBank. Pairwise comparison between *A. xishuangbannaensis* n. sp. and the other species of Cosmocercidae regarding the 18S sequences available in GenBank, including *Cosmocerca simile* (MN839758–MN839760), *Cosmocercoides dukae* (FJ516753), *C. pulcher* (LC018444, MH178322–MH178326), *C. qingtianensis* (MH032769–MH032771, MH178319–MH178321), *C. tonkinensis* (AB908160), *C. wuyiensis* (MK110872), *Nemhelix bakeri* (DQ118537) and *Raillietnema* sp. (DQ503461), displayed 1.88–3.77% nucleotide divergence.

#### Partial ITS-1 region

Three ITS-1 sequences of *A. xishuangbannaensis* n. sp. (accession numbers MW329035–MW329037) obtained were all 554 bp long, representing only one genotype. There are two species of *Aplectana* with ITS sequences available in GenBank, including *A. chamaeleonis* (MN907375‒MN907378) and *Aplectana* sp. '*Neyraplectana*' PNLS-530 (MH836325). Pairwise comparison between *A. xishuangbannaensis* n. sp. and the previously mentioned taxa showed 46.67 and 45.47% nucleotide divergence, respectively. Pairwise comparison between *A. xishuangbannaensis* n. sp. and the other species of Cosmocercidae regarding the ITS sequences available in GenBank, including *Cosmocerca japonica* (LC052772‒LC052782), *C. longicauda* (MG594349‒MG594351), *C. ornata* (MT108302), *Cosmocerca* sp. LL-2020 (MT108303), *C. simile* (MN839761‒MN839768), *Cosmocercoides pulcher* (MH178314–MH178318, LC018444), *C. qingtianensis* (MH178311–MH178313, MH032772–MH032774), *C. tonkinensis* (AB908160, AB908161) and *C. wuyiensis* (MK110871), displayed 28.53–47.52% of nucleotide divergence.

#### Partial 28S region

Three 28S sequences of *A. xishuangbannaensis* n. sp. (accession numbers MW329038–MW329040) obtained were all 740 bp long, representing only one genotype. There is only one species of *Aplectana*, *Aplectana* sp. '*Neyraplectana*' PNLS-530, with 28S sequence data (MH909070) available in GenBank. Pairwise comparison between *A. xishuangbannaensis* n. sp. and the previously mentioned taxon showed 20.67% of nucleotide divergence. Pairwise comparison between *A. xishuangbannaensis* n. sp. and the other species of Cosmocercidae with 28S sequences available in GenBank, including *Cosmocerca simile* (MN839755–MN839757), *Cosmocercoides pulcher* (LC018444) and *C. tonkinensis* (AB908160), displayed 16.78–17.94% of nucleotide divergence.

#### Partial *cox*1 region

Three *cox*1 sequences of *A. xishuangbannaensis* n. sp. (accession numbers MW327586–MW327588) obtained were all 384 bp long, representing only one genotype. There is no species of *Aplectana* with *cox*1 sequence registered in GenBank. Pairwise comparison between *A. xishuangbannaensis* n. sp. and the other species of Cosmocercidae regarding the *cox*1 sequences available in GenBank, including *C. japonica* (LC052756‒LC052770), *C. ornata* (MT108304), *Cosmocerca* sp. LL-2020 (MT108305), *C. simile* (MN833301‒MN833303), *C. pulcher* (MH178306–MH178310, LC052771) and *C. qingtianensis* (MH178303–MH178305, MH032775–MH032777), displayed 10.23–21.09% nucleotide divergence.

### Phylogenetic analyses

**Fig. 3:**
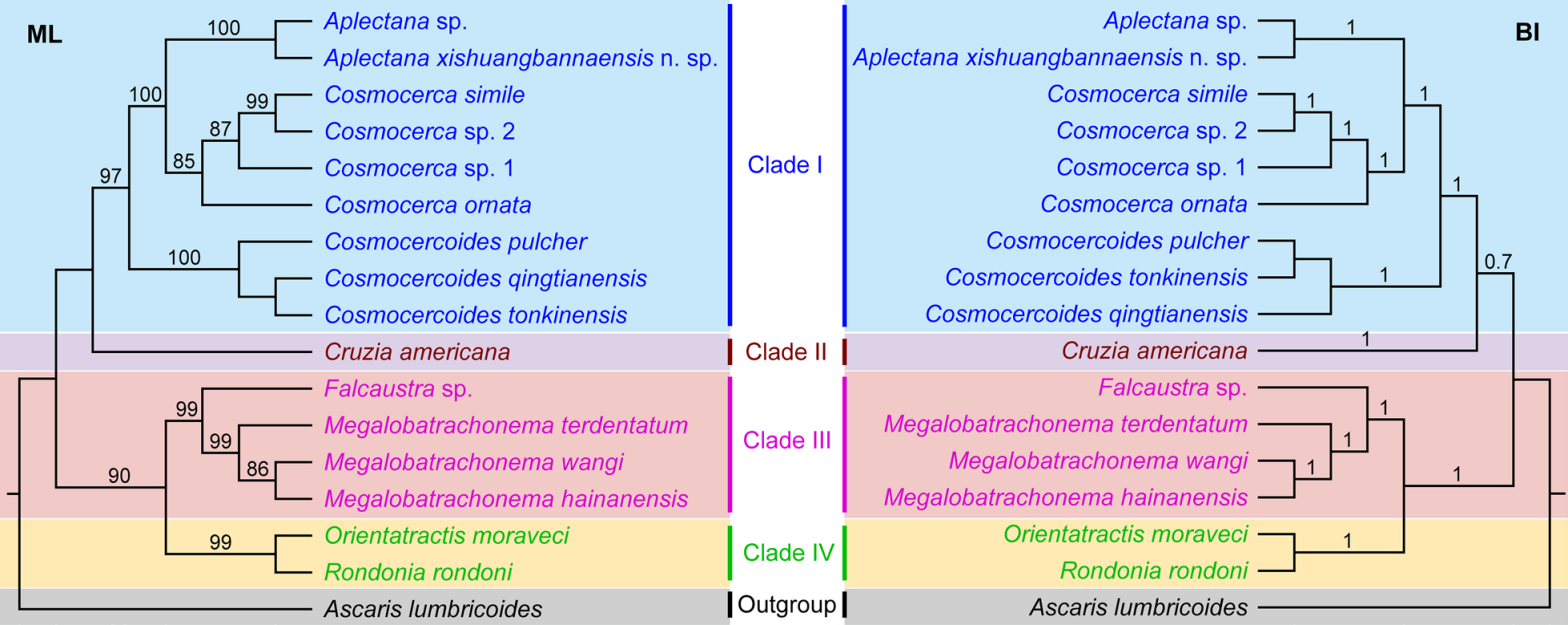
Maximum likelihood (ML) inference and Bayesian inference (BI) based on the 18S + 28S sequences of the rDNA showing the phylogenetic relationships of representatives of Cosmocercoidea. *Ascaris lumbricoides* Linnaeus, 1758 (Ascaridida: Ascaridoidea) was chosen as the outgroup. Bootstrap values exceeding 70% are shown

Phylogenetic trees inferred from maximum likelihood (ML) and Bayesian inference (BI) showed that representatives of Cosmocercoidea were divided into four major clades (Fig. 3). Clade I included the species of three genera *Cosmocerca*, *Cosmocercoides* and *Aplectana*, representing the family Cosmocercidae. Among the three genera, *Cosmocerca* displayed a closer relationship to *Aplectana* rather than *Cosmocercoides*. Clade II included only *Cruzia americana* (a common nematode parasite in the digestive tract of opossums), which belongs to the subfamily Cruzinae in the family Kathlaniidae according to the current classification [1]. Clade III included species of *Falcaustra* and *Megalobatrachonema*, which represent the family Kathlaniidae. The representatives of *Orientatractis* and *Rondonia* formed Clade IV, representing the family Atractidae.

## Discussion

The genus *Aplectana* (Cosmocercoidea: Cosmocercidae) is a group of zooparasitic nematodes, with approximately 50 nominal species mainly parasitic in various amphibians, and rarely occurring in reptiles worldwide [4, 5, 20–22]. The absence of rosette papillae or plectanes in males and presence of somatic papillae, lateral alae and two prodelphic ovaries, uteri containing numerous eggs of normal size in females, allocate the present specimens to the genus *Aplectana*. To date, only four species of *Aplectana* have been reported in China, namely *A. hainanensis* Bursey, Goldberg & Grismer, 2018, *A. hylae* Wang, 1980, *A. macintoshii* (Stewart, 1914) and *A. paucipapillosa* Wang, 1980 [22–24]. Lacking a gubernaculum, the new species can be easily distinguished from the four above-mentioned species (the four species all possessing a gubernaculum) [20, 22, 23].

In the genus *Aplectana*, *A. akhrami* (Islam, Farooq & Khanum, 1979), *A. artigasi* Puga & Torres, 1997, *A. chilensis* Lent & Freitas, 1948, *A. crossodactyli* Baker, 1980, *A. crucifer* Travassos, 1925, *A. delirae* (Fabio, 1971), *A. dubrajpuri* Sou & Nandi, 2015, *A. hoplobatrachusia* Sou, Sow & Nandi, 2018, *A. meridionalis* Lent & Freitas, 1948, *A. papillifera* (Araujo, 1977), *A. praeputialis* (Skrjabin, 1916), *A. tarija* Ramallo, Bursey & Goldberg, 2007, and *A. vercammeni* Le Van Hoa, 1962, have no gubernaculum [20, 22, 25–35], similar to the new species.

*Aplectana xishuangbannaensis* n. sp. differs from *A. dubrajpuri* and *A. meridionalis* in the different position of the excretory pore (situated at anterior end of oesophageal bulb *vs* at 1/2 between nerve ring and oesophageal bulb in the latter two species). With only one pair of precloacal papillae, *A. tarija*, which has six pairs of precloacal papillae, can be easily differentiated from the new species. *Aplectana artigasi*, *A. chilensis*, *A. crucifer*, *A. praeputialis*, *A. vercammeni* and *A. hoplobatrachusia* differ from *A. xishuangbannaensis* n. sp. by having relatively longer spicules (spicules representing 9.10–15.2% of body length in the former species *vs* spicules representing 5.98–7.09% of body length in *A. xishuangbannaensis* n. sp.). *Aplectana papillifera* can be easily distinguished from the new species by having a larger female body (5.90–8.50 *vs* 3.54–3.86 mm in *A. xishuangbannaensis* n. sp.), relatively shorter female tail (representing 4.47‒5.59% of body length in *A. papillifera vs* representing 9.78‒11.1% of body length in the new species) and a different arrangement and number of caudal papillae (precloacal: 10 pairs; paracloacal: 1‒2 pairs; postcloacal: 8 pairs in the former *vs* precloacal: 6 pairs; paracloacal: 3 pairs; postcloacal: 4 pairs in *A. xishuangbannaensis* n. sp.).

The new species differs from *A. crossodactyli* by having relatively longer spicules (*vs* representing 3.78–4.64% of body length in *A. crossodactyli*) and fewer precloacal papillae (6 pairs in the new species *vs* 20 pairs in the latter). *Aplectana xishuangbannaensis* n. sp. can be easily distinguished from *A. akhrami* by having a different position of the vulva (vulva from anterior extremity at 44.8–54.5% of body length in the new species *vs* vulva from anterior extremity at 29.0–30.6% of body length in *A. akhrami*) and a much longer female tail (*vs* 0.16 mm, representing 4.44–5.33% of body length in *A. akhrami*).

Currently, the specific diagnosis of *Aplectana* spp. remains based on morphology, and the genetic data of these parasites are severely limited. Based on the genetic analysis of *A. xishuangbannaensis* n. sp., no intraspecific nucleotide differences in 18S, ITS-1, 28S and *cox*1 regions among different individuals were noted, but a high level of interspecific genetic variation in these regions among species of the other genera in the Cosmocercidae was clear.

Our phylogenetic results are largely congruent with the traditional classifications of the Cosmocercoidea, which have been proposed based on morphological characters and ecological traits, including the structure of the oesophagus, the presence or absence of a precloacal sucker, the morphology of caudal papillae, the morphology of female reproductive organs and the reporductive strategies [1, 2, 36].

The systematic position of the subfamily Cruziinae has long been under debate. Our molecular phylogenetic results conflicted with the traditional classfication [1, 5, 40–42], which suggested that the subfamily Cruziinae should be moved out from the hitherto-defined family Kathlaniidae and elevated to a separate family. The highly specialized structure of the pharynx (the presence of unique pharyngeal lamellae) and the unique digestive system (the presence of an intestinal caecum) of this group support its full family status [43]. However, a more rigorous molecular phylogenetic study with broader representatives of the Cruziinae using different nuclear and/or mitochondrial genetic markers is required to further ascertain its systematic position.

The Cosmocercidae currently includes about 200 nominal species allocated in more than 20 genera, representing the largest family within Cosmocercoidea [1, 3, 21, 44]. However, the phylogenetic relationships among genera within Cosmocercidae is poorly understood because of the lack of genetic data. According to Chabaud (1978) [1] and Gibbons (2010) [44], the morphology of caudal papillae in males is one of the most important characters for generic diagnosis in the Cosmocercidae. Species of the genus *Aplectana* have no modified papillae (plectanes and/or rosette papillae), but those of *Cosmocerca* and *Cosmocercoides* have this character. Wilkie (1930) [45], Skrjabin et al. (1961) [5] and Chabaud (1978) [1] considered these genera with modified papillae more closely related to each other than *Aplectana*. However, our results indicated that *Cosmocerca* is closer to *Aplectana* rather than *Cosmocercoides*, conflicting with the traditional systematics based on morphology.

## Conclusions

The present study provided a preliminary molecular phylogenetic framework for the superfamily Cosmocercoidea based on 18S + 28S sequence data for the first time to our knowledge. The family Kathlaniidae is not a monophyletic group. Cruziidae is probably valid as a family for Cosmocercoidea. The genus *Aplectana* is closer to *Cosmocerca* than to *Cosmocercoides*, which is basal within Cosmocercidae. Moreover, morphological and genetic evidence both supported the hypothesis that our nematode specimens collected from *P. megacephalus* represent a new species of *Aplectana*, which is the fifth species in the genus reported from China. However, the phylogenetic aspects of Cosmocercoidea are far from being well understood.

## Data Availability

The nuclear and mitochondrial DNA sequences of *Aplectana xishuangbannaensis* n. sp. obtained in this study were deposited in GenBank database under the accession numbers MW329041–MW329043 (*18S* sequences), MW329035–MW329037 (*ITS-1* sequences), MW329038–MW329040 (*28S* sequences) and MW327586–MW327588 (*cox1* sequences). Type specimens of the new species were deposited in the College of Life Sciences, Hebei Normal University, Hebei Province, under the accession numbers HBNU–N-2020A009–11L, China.

## Abbreviations

SEM: Scanning electron microscopy
PCR: Polymerase chain reaction
ML: Maximum likelihood
BI: Bayesian inference
18S: Small subunit ribosomal DNA
28S: Large subunit ribosomal DNA
ITS: Internal transcribed spacer
*cox*1: Cytochrome *c* oxidase subunit 1
dl: Dorsal lip
vl: Ventrolateral lip
p: Large double cephalic papillae
pp: Paracloacal papilla
sp: Single median precloacal papilla
